# Multimodal knowledge expansion widget powered by plant protein phosphorylation database and ChatGPT

**DOI:** 10.3389/fbinf.2025.1687687

**Published:** 2025-10-15

**Authors:** Chunhui Xu, Yang Yu, Govardhan Khadakkar, Jiacheng Xie, Dong Xu, Qiuming Yao

**Affiliations:** 1 Bond Life Sciences Center, University of Missouri-Columbia, Columbia, MO, United States; 2 Institute for Data Science and Informatics, University of Missouri-Columbia, Columbia, MO, United States; 3 Department of Electrical Engineering and Computer Science, University of Missouri-Columbia, Columbia, MO, United States; 4 School of Computing, University of Nebraska Lincoln, Lincoln, NE, United States; 5 Nebraska Center for Virology, University of Nebraska, Lincoln, NE, United States

**Keywords:** multimodality, large language mode, plant protein phosphorylation, information retrieva, pathway identification

## Abstract

Biological databases are essential for providing curated knowledge, but their rigid data structures and restrictive query formats often limit flexible and exploratory user interactions. In the field of plant phosphorylation, manually curated and reviewed data represent only a small portion of the available knowledge, and users often seek information that goes beyond what is provided in structured databases. While large language models (LLMs) like ChatGPT-4o possess extensive contextual knowledge, integrating this capability into bioinformatics tools remains an open challenge. Here, we present a multimodal question-answering widget that integrates ChatGPT-4o with our Plant Protein Phosphorylation Database (P3DB). This system supports natural language queries and dynamic prompt formulation, enabling users to explore phosphorylation events, kinase-substrate relationships, and protein-protein interactions through a global entry. In another application, the widget leverages ChatGPT’s image interpretation functionality to extract regulatory pathways and phosphorylation markers from complex scientific figures. To build this widget effectively, we have explored multiple prompt strategies, including one-step, two-step, few-shot, and image-cropping techniques, demonstrating their impact on output accuracy and consistency. In addition, recent multimodal LLMs such as ChatGPT-5 and Gemini 1.5 have demonstrated comparable capabilities and adaptability when applied to our test cases and the developed widgets. Together, our application widget and results highlight the development of the ChatGPT-P3DB integration as a system that enhances user accessibility, enables visual extraction, and extends the current utility of biological knowledgebases through a flexible and adaptive framework. Our “ChatGPT-P3DB” is open-source and can be accessed on GitHub (https://github.com/yao-laboratory/p3db-chat). The frontend interface, “P3DB askAI” web module, can be accessed freely through https://www.p3db.org/ask-ai.

## 1 Introduction

Artificial intelligence (AI) and natural language significantly enhance content retrieval from databases by offering intuitive and user-friendly query interfaces (Reshma and Remya, 2018; Choi et al., 2021; Ye et al., 2023; Li and Jobson, 2024; Park et al., 2024). In bioinformatics, ChatGPT and other Large Language Models (LLMs) have been extensively utilized for diverse tasks, including knowledge extraction in genomic variants (Lu and Cosgun, 2025), gene set function annotations (Hu et al., 2025; Wang et al., 2025), medical data analysis (Chen et al., 2024a), and interactive reasoning in biomedicine (Wang et al., 2024a) and plant biology (Zhang et al., 2025). Particularly in building bioinformatics infrastructures and knowledge bases, ChatGPT can facilitate data accumulation from online resources (Blum et al., 2025; Pop et al., 2025). There are several attempts to build databases and knowledge foundations using ChatGPT, such as a microRNA and disease association database (Wang et al., 2024c), and an integrated Dietary Supplement Knowledgebase 2.0 (iDISK2.0) (Hou et al., 2025). InterPro, a protein family database, applied the GPT-4 model to generate descriptions and annotations (Blum et al., 2025). Similarly, Reactome prototyped a ChatGPT-assisted curation process for pathway annotations (Tiwari et al., 2023). However, the data harvested through LLMs and generative AI models typically raises concerns (Pop et al., 2025), and requires substantial manual verification before integration into curated databases or necessitates additional fine-tuning and re-training to align with existing domain-specific knowledge. For instance, using ChatGPT to gain gene interaction knowledge, will require stringent benchmarks (Chen et al., 2024b). Meanwhile, the multimodal capabilities of GPT-4 introduce new opportunities for bioinformatics applications, within knowledgebase environment. For example, GPT-4V has been tested to enable more advanced interpretation and understanding of scientific images within bioinformatics contexts (Wang et al., 2024b). This functionality holds promise to extend regular Optical Character Recognition (OCR) processes in bioinformatics in pathway identification and analysis (Shin and Pico, 2023). Nevertheless, ChatGPT is a good supplement to data collection methods for bioinformatic databases.

Rather than following the conventional paradigm of “ChatGPT for knowledgebases”, where LLMs are primarily used for automated data collection, our approach strengthens this relationship by adding a “knowledgebase for ChatGPT” linkage. We introduce a widget application that actively couples ChatGPT with a specialized plant phosphorylation database, creating a two-way interaction where the database not only supports but also enhances ChatGPT’s performance. This integration allows the knowledgebase to guide, correct, and optimize user prompts while serving as a validation layer for the model’s responses. By using GPT-4’s multimodal capabilities, the system supports natural language queries and scientific figure interpretation within the same framework. The result is an enriched user experience where ChatGPT becomes a centralized and interactive entry point, augmented by curated domain knowledge, to deliver complementary insights and extend the utility of structured bioinformatics data.

This synergy enhances the utility of the database without compromising or contaminating the integrity of existing curated data. Moreover, a knowledge base can play a crucial role in validating outputs from natural language searches, optimizing query performance, and guiding prompt generation for domain-specific tasks. Such an approach is particularly beneficial for databases that require extended periods for manual updates or for those lacking comprehensively structured existing knowledge.

Our team has been developing the Plant Protein Phosphorylation Database (P3DB) extensively (Yao et al., 2012; Yao et al., 2014), making it an ideal platform or testing bed to demonstrate this innovative framework through ChatGPT-P3DB coupled extension widgets. Several factors make P3DB especially suitable for this test case. First, although protein phosphorylation plays a central role in plant physiology and cell signaling cascade (Heintz et al., 2004; Jiang et al., 2021; Wang et al., 2021). But plant phosphorylation data are considerably less abundant compared to those available for mammalian systems; thus, users frequently seek additional insights when curated database searches yield limited results. Second, to maintain user trust and ensure data integrity, we deliberately avoid directly integrating LLM-generated content into P3DB. Instead, P3DB provides APIs that enhance ChatGPT’s functionality while keeping the two systems clearly separated, preserving the database’s role as a source of experimentally validated information. Third, publications processed behind ChatGPT are often more current and advanced than those reviewed manually for database inclusion. It’s known by our developers that expanding the data in P3DB takes serious human efforts. Under these circumstances, P3DB effectively guides ChatGPT prompts, while ChatGPT simultaneously enriches and extends the reach of P3DB, creating a complementary and mutually beneficial coupling.

## 2 Results

### 2.1 Overview of the system

Our core system is implemented in Python, designed to run on a Linux command line environment, and functions primarily as a backend service interacting with users through Application Programming Interface (API) endpoints. The system takes advantage of both the OpenAI API and our internally developed P3DB API to enable seamless coupling and enhance query precision. It comprises two main applications: one facilitating open-ended user queries related to plant phosphorylation, and another enabling the extraction and exploration of pathway knowledge from images containing gene and phosphorylation information. The first application employs the P3DB API to standardize and refine arbitrary user queries, generating targeted prompts. The second application leverages the P3DB API to create optimized, structured prompts, linking user-provided pathway images to carefully curated phosphorylation data. This system takes advantage of the capability of text reasoning and multimodality of ChatGPT-4o. By replacing the API in the future, this system is ready to support new ChatGPT models.

**FIGURE 1:**
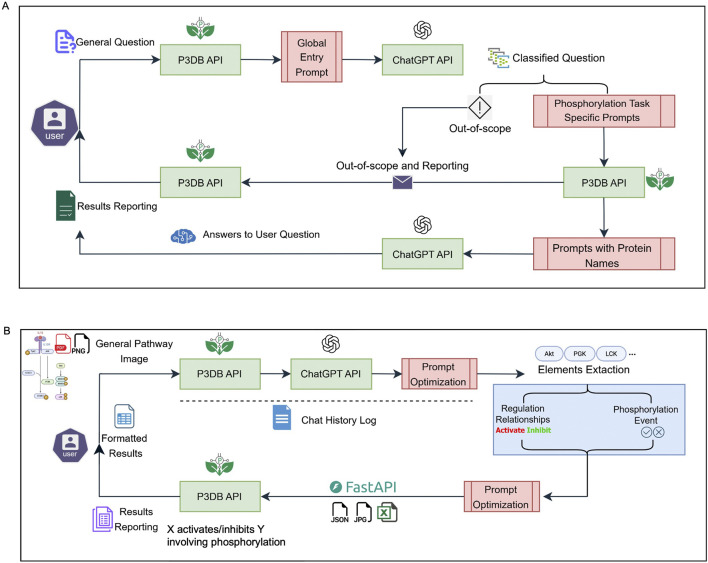
System overview of API-based user applications. **(A)** General question-answering system for phosphorylation-related queries. **(B)** Pathway extraction system for interpreting phosphorylation events from images. Green boxes indicate backend APIs; red boxes represent engineered and normalized prompts.

The first widget application (Figure 1A) handles general user questions regarding plant phosphorylation. When a user submits a query, the system initially uses the P3DB API to normalize and refine the question by going through a global entry prompt. This prompt is sent to the ChatGPT API, which classifies the query and determines whether it falls within the scope of plant phosphorylation topics supported by P3DB. If the question is classified as out of scope, it is logged and reported, ensuring transparency and maintaining database integrity. For in-scope queries, the system utilizes phosphorylation-specific prompts, asks the user for confirmation or to provide revisions, and returns ChatGPT answers (with a generative AI warning to users) together with precise protein identity information and P3DB phosphorylation record link from our P3DB API. This global entry strategy provides flexible, detailed and reliable answers to the user, while avoiding any potential contamination of the authoritative P3DB data. In our experiments, we will also use P3DB data and API to enhance the usage of phosphorylation questions by recognizing protein IDs and substitutes by more effective names if possible.

The global entry prompt provides a flexible interface for handling open-ended user questions. To evaluate the effectiveness of this design, we tested 10 real-world cases derived from recent plant protein phosphorylation publications (Ding et al., 2020; Lin et al., 2020; Lin et al., 2022; Chen et al., 2021; Jiang et al., 2021; Wang et al., 2021; Li et al., 2022; Zhang and Zhang, 2022; Geng et al., 2024; Wei et al., 2024), formulating each case as a user question in arbitrary formats. The results demonstrated that our universal prompt design effectively managed all 10 scenarios within the plant science domain (Supplementary Table S1). We did not perform systematic or exhaustive testing for this step, as our system inherently supports user intervention through a human-in-the-loop approach, allowing users to review and correct the normalized questions if needed. Currently, our framework can support redirection of user questions into categories such as phosphorylation event identification (determining if a protein can be phosphorylated), kinase-substrate relationships (whether a specific kinase phosphorylates a given substrate), and protein-protein interaction (PPI) questions (Supplementary Figure S1). Questions falling outside these categories are appropriately classified as out-of-scope. This approach ensures that, at a minimum, unsupported or irrelevant queries are identified and prevented from compromising the quality and focus of our knowledge base. This global entry prompt is very expandable for future usage or for a different purpose through this question routing idea.

The second widget application (Figure 1B) focuses on extracting and analyzing biological pathway information from user-submitted images. Multimodal capability in the new version of ChatGPT-4o provides a more convenient way to implement this image-based functionality, which does not require the user or developer to have image processing skills. After receiving a pathway image, the system employs the ChatGPT API to interpret the content and perform initial model tuning to identify relevant elements, i.e., gene names, regulation types and directions, and the involvement of phosphorylation events. This will produce raw output structured in JSON format. This JSON output undergoes further element extraction, resulting in a clearly structured, table-formatted summary, which can be easily reviewed by users. Subsequently, the identified gene or protein names can be cross-linked by P3DB’s curated phosphorylation data via API calls. P3DB provides different strategies to apply our pre-designed prompts to perform this image-based pathway extraction application. The detailed testing cases for this application will be described in the following sections.

### 2.2 Protein names are more appropriate for developing prompts

The global entry prompt normalizes users’ open-ended questions into specific phosphorylation-related queries. To ensure the effectiveness of these text-based prompts used in the first application, we performed targeted tests and optimizations on each prompt type individually, assisted by P3DB data.

**FIGURE 2:**
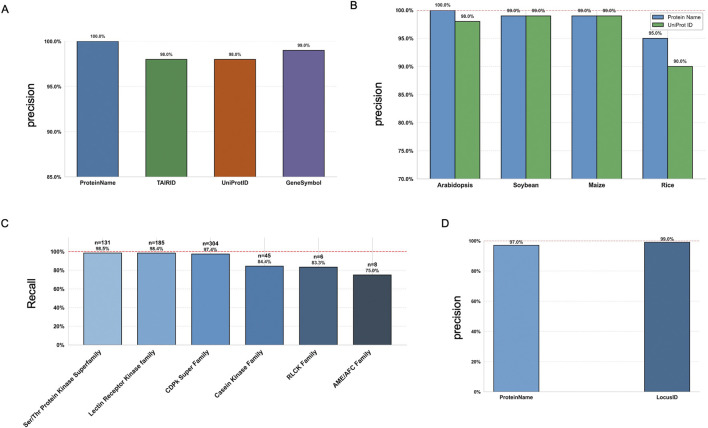
Phosphorylation-specific tasks and evaluation results using P3DB datasets and ChatGPT-4o API. **(A)** Precision scores for the “Is this protein phosphorylated?” task, evaluated using TAIR IDs, gene symbols, UniProt IDs, and full protein names. **(B)** Precision scores for the same task in major plant species (Arabidopsis, soybean, maize, rice), using UniProt IDs and full protein names from randomized P3DB data. **(C)** Precision scores across kinase families for the “Does this kinase phosphorylate the substrate?” task, using KiC-assay data from Arabidopsis. **(D)** Precision scores for Arabidopsis protein-protein interaction (PPI) questions, evaluated using both protein names and TAIR IDs.

First, we randomly selected 100 known phosphoproteins from Arabidopsis, using different identifiers, full Protein Names, TAIR IDs, UniProt IDs, and Gene Symbols, to evaluate the precision of phosphorylation event identification when formulating prompts for ChatGPT. The bar plot (Figure 2A) clearly illustrates the performance differences among these identification methods (Supplementary Table S2). Using the full protein name achieved the highest accuracy (100%), indicating it provides the most effective input for prompt formulation with ChatGPT. Gene Symbols also showed strong performance with 99% accuracy, closely followed by TAIR IDs and UniProt IDs, reaching 98% accuracy. Although the difference is not statistically significant, a similar trend was observed with ChatGPT-5 and Gemini 1.5, both of which showed slightly better performance when using full protein names and gene symbols as input identifiers (Supplementary Table S2; Supplementary Figures S2, S3). These results suggest that providing more explicit and descriptive protein information enhances ChatGPT’s ability to accurately interpret and respond to phosphorylation-related prompts. For other major plant species, i.e., soybean, maize, and rice, we observed similar trends (Figure 2B). We compared the precision by including UniProt IDs and full protein names in ChatGPT prompts (Supplementary Table S2). Across all tested species, full protein names consistently outperformed UniProt IDs, demonstrating better alignment with ChatGPT’s language understanding capabilities.

P3DB includes kinase-substrate relationship data generated from KiC-assay experiments (Ahsan et al., 2013; Ahsan et al., 2024; Jorge et al., 2024), which we used to evaluate prompt effectiveness across different kinase families in Arabidopsis. As shown in the figure, ChatGPT’s and Gemini’s ability to correctly classify kinases varies by family, with higher recall observed for families such as the S/T-related kinase superfamily, the Lectin Receptor Kinase family and CDPK family (Figure 2C; Supplementary Figures S2C, S3C; Supplementary Table S3), which are mostly studied in plant science domain. Kinase families with fewer cases may be disadvantaged due to sampling bias. In addition, P3DB contains a substantial collection of PPI data for Arabidopsis (Figure 2D; Supplementary Table S4). Since the quality and usability of the PPI dataset, as well as ChatGPT’s performance (GPT models) on similar PPI-related tasks, have been extensively reported in previous studies (Rehana et al., 2024; Chang et al., 2025), we did not perform additional ChatGPT-specific testing for PPI performance.

In addition, P3DB supports ID mapping API (see Methods), enabling seamless conversion of various identifiers such as UniProt IDs, TAIR IDs, and gene names. We utilized P3DB APIs to dynamically insert protein names into normalized user prompts, enhancing user experience with ChatGPT. This functionality exemplifies the strength of the ChatGPT-P3DB coupling, allowing accurate and context-aware query generation without compromising data integrity or user flexibility.

### 2.3 Phosphorylation-associated pathway identification from scientific images

Phosphorylation plays a central role in plant signaling pathways (Lin et al., 2020; Jiang et al., 2021; Wang et al., 2021; Zhang and Zhang, 2022), and many plant phosphorylation-related publications include complex figures that depict gene activations, inhibitions, and phosphorylation events. However, users often lack the image processing skills or tools to extract gene relationships and compare them with curated phosphorylation data in P3DB. To address this gap, the second application of our widget leverages the multimodal capabilities of ChatGPT-4o to interpret pathway diagrams and extract structured biological insights using different designs of image-based prompts (Supplementary Table S5).

For this prompt construction and testing, we manually curated 18 pathway images from 18 recent peer-reviewed plant phosphorylation publications published between 2019 and 2025 (Supplementary Table S6). Each image was manually annotated to establish ground-truth data, including gene pairs, regulatory types (activation or inhibition, direct or indirect), and phosphorylation involvement. We then evaluated two prompting strategies using ChatGPT-4o (also work in ChatGPT-5 and Gemini-1.5): a one-step method, which asks ChatGPT to identify all relevant information (gene pairs, regulation types, and phosphorylation events) in a “convenient” single prompt, and a two-step method, which separates the analysis into two refined steps. In the two-step approach, the first prompt extracts gene pairs and regulatory types, and the second prompt focuses specifically on identifying visual phosphorylation markers (e.g., small “P” circles) to determine whether each regulatory interaction involves phosphorylation (looping through identified interactions from the first step) (Supplementary Table S5).

**FIGURE 3:**
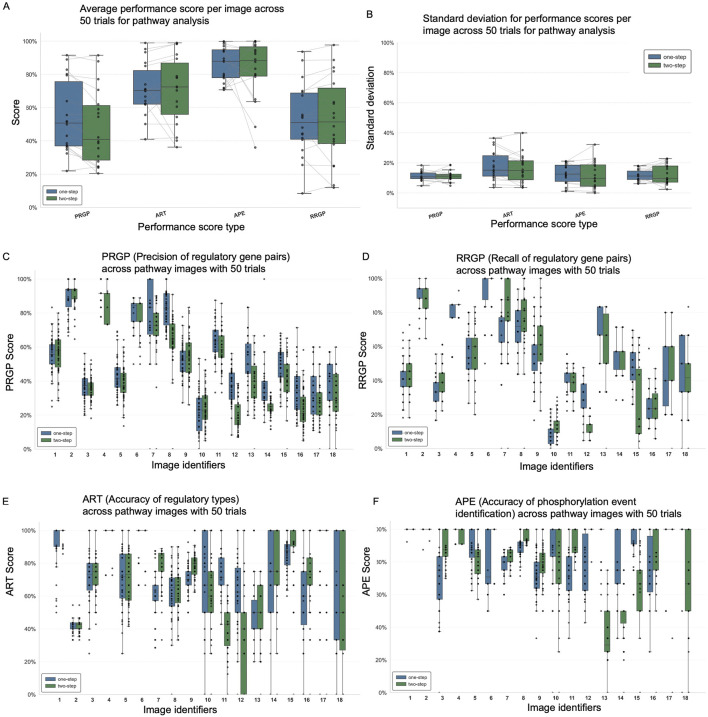
ChatGPT-4o image processing results for 18 pathway diagrams using one-step and two-step prompt approaches. **(A)** Average performance scores across all images comparing one-step and two-step prompt methods. **(B)** Standard deviation of performance scores for each method, indicating consistency. Box plots showing distribution across 50 trials for each image: **(C)** PRGP (precision of regulatory gene pairs), **(D)** RRGP (recall of regulatory gene pairs), **(E)** ART (accuracy of regulatory types), and **(F)** APE (accuracy of phosphorylation event identification).

Both one-step and two-step prompts performed reasonably well across all metrics (Figure 3A), including PRGP (Precision of Regulatory Gene Pairs), ART (Accuracy of Regulatory Types), APE (Accuracy of Phosphorylation Event), and RRGP (Recall of Regulatory Gene Pairs). The two-step method demonstrated marginally higher performance in APE but lower in PRGP. While the performance gains were modest, given that the prompts are separated, the two-step strategy allows for more targeted extraction and interpretation of phosphorylation-specific features within complex diagrams. APE benefited the most from the two-step refinement, confirming that separate attention to phosphorylation markers improves accuracy in phosphorylation event detection. In addition to accuracy, we evaluated consistency using standard deviation across the 18 samples (Figure 3B). The two types of prompts display a variation of around 10%–20% among experimental repeats.

The detailed performance breakdown across individual images reveals that the visual complexity and layout of pathway diagrams can significantly impact ChatGPT’s ability to accurately extract information (Figure 3C–F; Supplementary Tables S7, S8). For example, Image 12 exhibited consistently poor performance across all evaluated metrics, likely due to its highly crowded layout and dense visual elements, which make it challenging for the model to identify gene relationships and phosphorylation events all at once. Some images also contain gene names or relationships distant from the marked phosphorylation events. These results highlight the sensitivity of multimodal prompt performance to image design and suggest that tailored strategies may be needed for more complex figures. The same images and prompts were also tested in two other multimodal LLMs, i.e., ChatGPT-5 and Gemini-1.5, and we observed similar performance patterns on challenging images 3,5,10,12,14 (Supplementary Figures S4, S5; Supplementary Tables S9, S10). In the following section, we explore additional prompt engineering techniques and design considerations that may improve performance on visually challenging images.

### 2.4 Additional practical strategies or techniques for pathway identification

#### 2.4.1 Image cropping test

**FIGURE 4:**
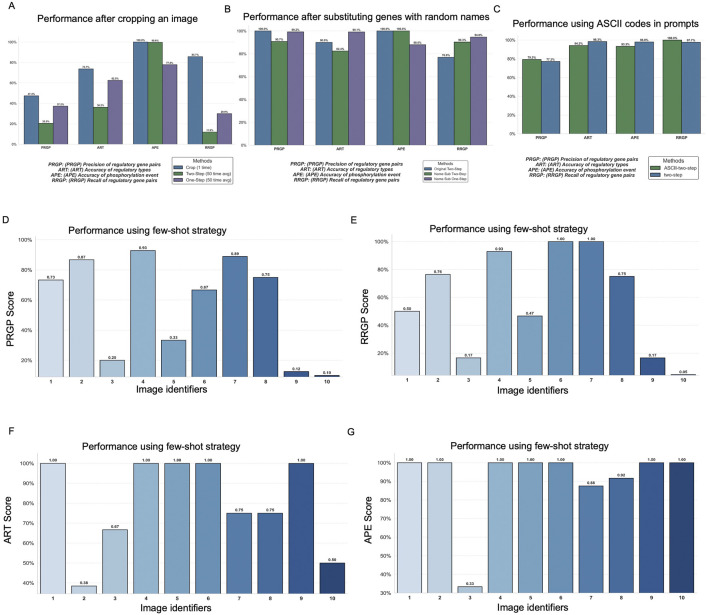
Analyses exploring image and prompt design and engineering strategies for image-based pathway extraction by ChatGPT-4o. **(A)** Comparison of full images versus cropped, segmented images for improving extraction accuracy. **(B)** Evaluation of original images versus images with gene names substituted by random alphabetic labels, testing robustness to semantic information. **(C)** Comparison of standard prompts with prompts augmented using ASCII formatting. Performance comparison between zero-shot (original) prompts and few-shot prompts using example-guided prompting strategies, **(D)** PRGP (precision of regulatory gene pairs), **(E)** RRGP (recall of regulatory gene pairs), **(F)** ART (accuracy of regulatory types), and **(G)** APE (accuracy of phosphorylation event identification).

To address the poor performance observed in visually dense diagrams (i.e., in Image 12), we conducted an image cropping test to isolate smaller, less crowded segments of the figure. The results (Figure 4A; Supplementary Table S11) show that performance improves by 10%–60% on different scores when cropped images are used. Across all metrics, the cropped version outperformed both the original one-step and two-step methods. This confirms that clutter and visual complexity in original figures hinder ChatGPT’s ability to extract biological relationships, and that segmenting complex figures can serve as a practical strategy to improve multimodal interpretation. Given the variability across images, image cropping may not yield consistent and robust gains among all cases.

#### 2.4.2 Substitute with nonsense names

In this experiment, we tested whether substituting actual gene names with meaningless, random alphabetic identifiers (e.g., “ABC”, “XYZ”) would impact ChatGPT’s ability to parse and analyze the pathway information. The substitution had a moderate impact on performance scores, typically ranging from 10%–20% variation across different metrics. These effects were inconsistent, sometimes increasing and sometimes decreasing performance, and were observed across both one-step and two-step prompting methods, generally falling within the range of standard deviations reported previously. This suggests that ChatGPT’s interpretation of pathway diagrams is driven primarily by the structural and visual features of the image rather than its reliance on domain-specific knowledge or learned associations from real-world gene names (Figure 4B; Supplementary Table S12). In other words, the model’s visual understanding is limited enough to function without textual priors from the literature.

#### 2.4.3 ASCII used in prompts

Some prior research (Jiang et al., 2024) has suggested that injecting random noise characters or using ASCII-art formatting can enhance ChatGPT’s robustness in cross-modal tasks. Motivated by this observation, we tested the impact of including ASCII-style text at the beginning of our prompt to assess whether it could enhance ChatGPT’s performance in our image-based pathway extraction task. However, as shown in our results (Figure 4C; Supplementary Table S13), the ASCII-enhanced prompts did not yield any noticeable improvement. In fact, in some cases, standard (non-ASCII) prompts slightly outperformed the ASCII versions. These findings suggest that, in the specific context of interpreting biological pathway images, ASCII formatting offers no substantial advantage in ChatGPT4o. Therefore, future prompt optimization efforts should prioritize strategies like image segmentation and visual layout enhancement rather than relying on syntactic tricks or formatting noise.

#### 2.4.4 Few-shot (two-shot) testing

To evaluate the potential benefits of few-shot learning in the context of image-based prompt design, we conducted a two-shot testing experiment. Few-shot prompting is a commonly used technique to enhance the performance of LLMs by providing a few relevant examples. In our test, images numbered 1 through 10 were used as the primary evaluation set, while images 11 and 12, both rich in diverse pathway content, served as embedded few-shot examples within the prompts.

The results showed varied impacts across performance metrics (Figure 4D–G; Supplementary Table S14). For instance, specific images that previously performed poorly, such as images 9 and 10, exhibited marginal improvements in metrics like ART and APE. Conversely, some images, such as image 3, continued to demonstrate consistently low performance, even under the few-shot settings.

This variability suggests that while well-structured and information-rich few-shot examples can help in some cases, they do not consistently enhance model performance across all image types, displaying context sensitivity. In summary, incorporating few-shot prompts provided limited benefit for complex and visually dense scientific figures in our domain. Additional work may be required to tailor few-shot designs to specific image types or layout characteristics for more reliable improvement.

### 2.5 Integrate the system into the P3DB web interface

The ChatGPT-P3DB web interface, named P3DB-AskAI, offers an integrated platform that supports two primary functionalities: (a) natural language-based general querying and (b) image-based pathway interpretation. These modes are accessible through a user-friendly sidebar panel, allowing simple toggling between text and image input workflows. Each module includes a brief interactive tutorial and example to guide users through its features, enabling intuitive engagement with phosphorylation-related tasks.

**FIGURE 5:**
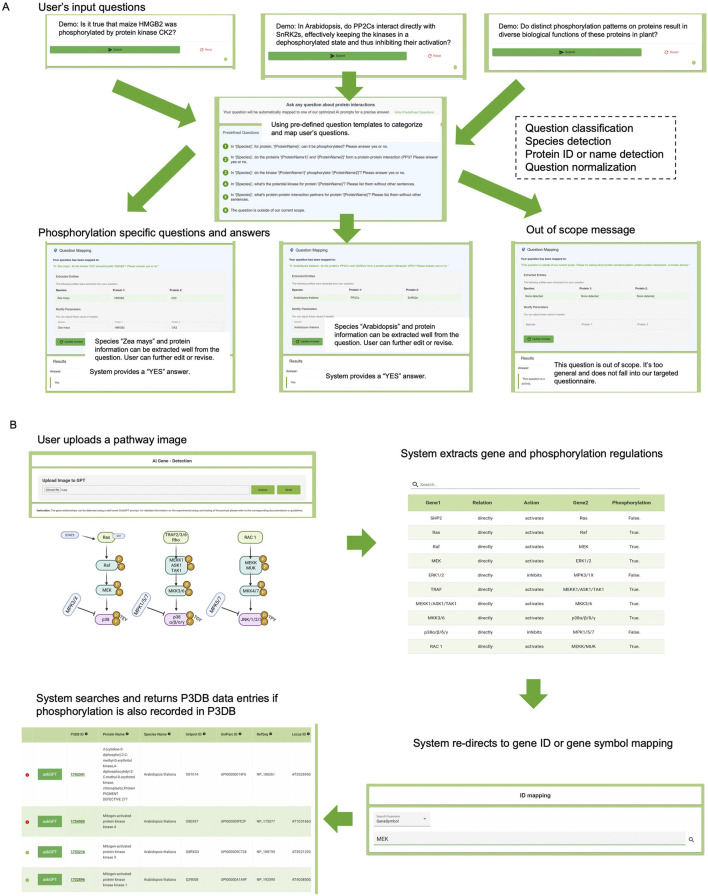
P3DB-AskAI web interface demonstration. **(A)** A user’s general input question is automatically normalized into a phosphorylation-specific query or flagged as out of scope. **(B)** A user-uploaded pathway image is processed to identify gene relationships and phosphorylation-regulated interactions, which are then linked to curated gene and phosphorylation data in P3DB.

The natural language query interface is designed to accept a wide range of user questions, providing a flexible and universal interaction experience (Figure 5A). Users begin by entering free-form questions related to plant phosphorylation. The system automatically performs species detection, protein name or ID recognition, and query classification. Based on the content, the interface either normalizes the input into one of several supported phosphorylation-specific tasks, such as determining whether a protein is phosphorylated, identifying kinase-substrate relationships, or detecting protein-protein interactions, or returns an “out-of-scope” message when the query cannot be addressed by the underlying database. Once a question is classified, the system responds with relevant, curated data from P3DB, improving accessibility to high-quality biological insights without requiring a structured query language or prior database expertise. For involving humans in the loop, we have designed an additional interface to allow users to correct species or protein names if the system fails to do the normalization.

In the image-based pathway extraction workflow (Figure 5B), users upload a pathway diagram, often derived from scientific figures. The system parses the image using ChatGPT-4o′s multimodal capabilities, extracting gene names, interaction types (activation/inhibition), and phosphorylation events. These extracted relationships are displayed in a tabular format, making the visual content computationally accessible. In addition to the command line version, the web system supports redirection through gene ID or gene symbol mapping, and then queries P3DB to cross-reference whether the identified proteins and phosphorylation events are supported by experimental evidence. Matched entries are presented with detailed annotation and links to original P3DB records. This provides a connection between the external information and the internal P3DB curations.

## 3 Discussion

Our work demonstrates the feasibility and benefits of integrating ChatGPT with a domain-specific bioinformatics resource, P3DB, to support flexible, user-driven query interpretation and pathway analysis. Although we did not retrain the GPT models, previous studies have shown that prompt engineering and retrieval-augmented generation (RAG)-based systems can perform effectively in various bioinformatics applications, such as gene variant interpretation and gene set annotation, by leveraging pre-trained ChatGPT models (Hu et al., 2025; Lu and Cosgun, 2025; Wang et al., 2025).

Similar to a network router, our design of a global entry prompt offers a user-friendly and adaptable interface that accepts open-question input and converts it into task-specific prompts, such as phosphorylation event recognition, kinase-substrate interaction, and protein-protein interaction questions. This normalization step bridges the gap between general user queries and structured database queries, enabling more accessible interaction with complex biological data from the public domain to a professional knowledgebase.

One key finding is that full protein names outperform other identifiers (e.g., TAIR IDs, UniProt IDs, and gene symbols) when used in prompt construction. This may be due to ChatGPT’s stronger language modeling capabilities with descriptive text, as full names likely contain richer semantic cues that align with its pretrained knowledge. This insight can inform the design of user interfaces and APIs, suggesting that mapping abbreviated identifiers to full names before querying consistently yields better performance.

In parallel, our image-based application highlights the importance of visual pathway interpretation as a complement to text-based database queries. Many critical insights in plant phosphorylation research are encoded in figures that contain multiple signaling pathways, regulation types, and phosphorylation markers. Our system, powered by ChatGPT-4o′s multimodal capabilities, successfully extracts structured biological relationships from these images. The use of refined prompting strategies (e.g., two-step and few-shot prompts) further improved both the accuracy and consistency of output, especially when analyzing visually complex diagrams. Image cropping and few-shot prompting emerged as practical techniques to address noisy or dense input, which can be further explored and automated.

In terms of system limitations, we observed that performance declines were most observed in crowded or visually complex pathway figures (such as image 12), where overlapping labels and overlapping graphical elements hindered major gene relationships and phosphorylation events (images 13 and 15). Ambiguous symbols, for example, unclear phosphorylation markers or inconsistent arrow styles, as well as misclassification of kinase-substrate relationships, were also common in low-performing cases. These cases highlight the importance of both prompt refinement and pre-processing strategies, such as image segmentation and image enhancement, to improve extraction performance in future work.

In this study, we primarily focused on ChatGPT-4o for both natural language and image-based prompt testing, tested minimally on ChatGPT-5 and Gemini-1.5. In the final system implementation and web deployment, our decision was driven by practical considerations, not necessarily by our limited benchmarks. By the time of writing, ChatGPT-4o currently offers convenient, economic, and efficient multimodal APIs, with strong support for text and image inputs, making it a suitable platform for testing end-to-end workflows in real-world biological tasks. Moreover, the methods and prompt designs explored in our work, such as global entry normalization, two-step prompting, and few-shot image interpretation, are model-agnostic by nature. They can be readily adapted to other LLMs in future work, by replacing the backend APIs (ChatGPT-5, Gemini-1.5 or future models), similar to the work of GeneAgent (Wang et al., 2025). Our goal was to establish a proof of concept using a stable and well-documented system coupling GPT and P3DB, focusing on system development instead of comparative evaluation and benchmark type of work. The user experience of extending P3DB search through ChatGPT is designed to go beyond strictly reviewed data, allowing for exploratory and complementary insights. As a result, the system is not burdened by the need to achieve extreme accuracy, but instead focuses on enhancing accessibility, flexibility, and knowledge discovery.

Together, these results showcase how the ChatGPT-P3DB coupling addresses two major gaps: (1) the lack of flexible natural language guided by a domain-specific knowledge base and (2) the difficulty of extracting structured knowledge from visual scientific content. This dual-capability system exemplifies the potential of AI-augmented knowledge bases to expand user accessibility, improve data integration, and streamline analysis workflows in plant biology and beyond. As AI technologies continue to evolve, coupling curated bioinformatics databases with powerful language models offers a scalable path toward more intelligent, multimodal biological knowledge systems. Looking forward, the framework presented in this manuscript can be extended to support other post-translational modifications (e.g., ubiquitination, acetylation) by adapting the database coupling and prompt design accordingly. Additionally, the integration of spatial omics images (Martinez-Val et al., 2021; Crook et al., 2022) or subcellular localization maps (Currie et al., 2024; Hein et al., 2025) could further expand the system’s multimodal capability to interpret biologically relevant functions from visual data.

## 4 Materials and methods

### 4.1 Data collection

The global entry prompt was tested using real scientific data sourced from peer-reviewed publications identified via a random search using the keyword “plant phosphorylation” (specific PubMed IDs are listed in the Supplementary Tables). Pathway images were similarly obtained through a general Google image search using the same keyword from scientific publications during 2019–2025. Figures were manually filtered to select those explicitly depicting multiple pathways, clear gene identifiers, regulatory interactions (such as inhibition and activation), and marked phosphorylation events.

Phosphorylated proteins used for prompt testing, particularly those from Arabidopsis, maize, soybean, and rice, were randomly retrieved from our internal P3DB database. In addition, PPI data and kinase-substrate relationship data derived from KiC-assay experiments (Kim et al., 2025) were obtained from P3DB. All protein identifiers and corresponding full names used in testing were also sourced from P3DB, with further methodological details provided in subsequent sections.

### 4.2 ID mapping

Our P3DB APIs for ID mapping were constructed using data obtained from Phytozome (Goodstein et al., 2012), EnsemblPlants (Bolser et al., 2017), and STRING (von Mering et al., 2003). Gene annotations, sequence headers, and UniProt ID mapping tables were extracted from these resources and stored in a hybrid database system combining MongoDB and MySQL.

### 4.3 Prompt precision calculation

For phosphorylation-associated prompts, we calculated only precision, as P3DB exclusively contains confirmed positive datasets. Generating a comprehensive negative dataset for phosphorylation events is challenging and may introduce artificial bias. Therefore, precision was defined as the proportion of correct positive predictions (i.e., “yes” answers) relative to the total number of predictions made.

To systematically evaluate the prompts, we utilized APIs from multimodal LLMs to test both the phosphorylation-related and image-based queries. Specifically, we used ChatGPT models gpt-4o-2024-08-06 and gpt-5-2025-08-07, along with the Gemini model gemini-1.5-pro, to ensure consistency and comparability.

### 4.4 Evaluation metrics for image-based pathway extraction

Following ChatGPT-4o documentation, our system supports standard image formats, including PNG and JPEG. In this study, pathway figures were captured via screenshot from peer-reviewed publications and saved in PNG or JPEG format. These images were then processed using structured prompts to extract pathway components and biological interactions. Evaluation metrics were kept consistent across different prompting strategies for valid comparisons. The following metrics were used to evaluate image interpretation performance: PRGP (Precision of Regulatory Gene Pairs), the proportion of gene pairs identified by the model as regulatory interactions that are truly correct, among all predicted gene pairs; RRGP (Recall of Regulatory Gene Pairs), the proportion of true regulatory gene pairs the model correctly identified, out of all ground-truth gene pairs; ART (Accuracy of Regulatory Types), the percentage of correctly predicted interaction types (e.g., activation or inhibition; direct or indirect) among all identified interactions; APE (Accuracy of Phosphorylation Event), the proportion of phosphorylation events correctly identified by the model, relative to the total number of predicted phosphorylation events.

Evaluation of regulatory types was performed only when the gene pair was correctly identified. Similarly, phosphorylation events were evaluated only when the regulatory type was correctly predicted. Ground-truth data were manually curated from each image (Supplementary Table S6). To match natural biological interpretation, we adopted the following guidelines. Elements placed above arrows were treated as gene names when they served a regulatory function; When two gene names appeared together in a single graphical object (e.g., a box), they were annotated as a composite gene entity due to ambiguous attribution. In alignment with standard biological diagram conventions, arrows were interpreted as activation, while T-bars were considered inhibitory interactions.

### 4.5 P3DB-askAI web development

The ChatGPT-P3DB application widget was developed and integrated into the P3DB web platform using the Angular framework and TypeScript, ensuring full compatibility with the existing architecture. This web-based implementation enhances user experience beyond that of a traditional command-line interface by offering an interactive and intuitive environment for both text-based and image-based queries. Users can access the tool directly from the P3DB homepage by navigating to the “Tools” menu and selecting “Ask AI” from the dropdown list. The application features two main modes: (a) natural language query processing and (b) pathway image analysis. Both modes are supported by P3DB backend APIs, as described in previous sections, which enable dynamic prompt generation, result retrieval, and protein ID normalization. It is also leveraged by the OpenAI API (gpt-4o-2024-08-06) and the ID mapping module described above.

## 5 Data and code availability

Our backend system “ChatGPT-P3DB” is open-source and can be accessed on GitHub (https://github.com/yao-laboratory/p3db-chat). The frontend interface, “P3DB askAI” web module, can be accessed freely through https://www.p3db.org/ask-ai. Our testing cases and results are summarized in the supplementary tables. The original data from existing publications are mentioned as PubMed IDs in supplementary tables. All prompts in this paper are listed in the supplementary table as well as on the GitHub code base. The system was tested on Python version 3.9 and OpenAI SDK version 1.107.2.

## Data Availability

The original contributions presented in the study are included in the article/Supplementary Material, further inquiries can be directed to the corresponding authors.
