# Supplementary material for: Multimodal knowledge expansion widget powered by plant protein phosphorylation database and ChatGPT
Source: Front Bioinform. 2025 Oct 15;5:1687687. doi: 10.3389/fbinf.2025.1687687 (PMC12568720; doi:10.3389/fbinf.2025.1687687)
Supplement: Supplementary file 15 [file DataSheet1.docx]

Multimodal Knowledge Expansion Widget Powered by Plant Protein Phosphorylation Database and ChatGPT

Supplementary Material

# Supplementary Data

N/A.

# Supplementary Figures and Tables

## Supplementary Figures (see PDF file)

## Supplementary Figure 1. Illustration of the global entry prompt using the ChatGPT-4o (OpenAI) web interface. General user queries are automatically normalized into phosphorylation-specific questions through prompt engineering.

**Supplementary Figure 2.** Phosphorylation-specific tasks and evaluation results using P3DB datasets and ChatGPT5 API. (A) Precision scores for the “Is this protein phosphorylated?” task. (B) Precision scores for the same task in major plant species (Arabidopsis, soybean, maize, rice), using UniProt IDs and full protein names from randomized P3DB data. (C) Precision scores across kinase families for the “Does this kinase phosphorylate the substrate?” task, using KiC-assay data from Arabidopsis. (D) Precision scores for Arabidopsis protein-protein interaction (PPI) questions, evaluated using protein names.

**Supplementary Figure 3.** Phosphorylation-specific tasks and evaluation results using P3DB datasets and Gemini API. (A) Precision scores for the “Is this protein phosphorylated?” task. (B) Precision scores for the same task in major plant species (Arabidopsis, soybean, maize, rice), using UniProt IDs and full protein names from randomized P3DB data. (C) Precision scores across kinase families for the “Does this kinase phosphorylate the substrate?” task, using KiC-assay data from Arabidopsis. (D) Precision scores for Arabidopsis protein-protein interaction (PPI) questions, evaluated using protein names.

**Supplementary Figure 4**. ChatGPT5 image processing results for 18 pathway diagrams using two-step prompt approach. (A) Distribution of average performance scores across all images by two-step prompt method. (B) Distribution of the standard deviation of performance scores for the two-step prompt. Box plots in (C-F) showing performance distribution across 10 trials for each image. (C) PRGP (precision of regulatory gene pairs), (D) RRGP (recall of regulatory gene pairs), (E) ART (accuracy of regulatory types), and (F) APE (accuracy of phosphorylation event identification).

**Supplementary Figure 5**. Gemini image processing results for 18 pathway diagrams using two-step prompt approach. (A) Distribution of average performance scores across all images by two-step prompt method. (B) Distribution of the standard deviation of performance scores for the two-step prompt. Box plots in (C-F) showing performance distribution across 10 trials for each image. (C) PRGP (precision of regulatory gene pairs), (D) RRGP (recall of regulatory gene pairs), (E) ART (accuracy of regulatory types), and (F) APE (accuracy of phosphorylation event identification).

## Supplementary Tables (see Excel files)

**Supplementary Table 1**. Global entry prompt for phosphorylation knowledge retrieval and testing cases.

**Supplementary Table 2**. Prompt-based retrieval for phosphorylation events on Arabidopsis, soybean, rice, and maize.

**Supplementary Table 3**. Prompt-based retrieval for kinase-substrate relationships on Arabidopsis from the Kic-assay network.

**Supplementary Table 4**. Prompt-based retrieval for protein-protein interaction relationships on Arabidopsis, by locusID and protein full name.

**Supplementary Table 5**. Image-based prompts to extract pathway information.

**Supplementary Table 6**. Testing cases for pathway extraction from images.

**Supplementary Table 7**. Two-step prompt-based image analyses over 18 plant phosphorylation pathway images (run ChatGPT-4o API 50 times).

**Supplementary Table 8**. One-step prompt-based image analyses over 18 plant phosphorylation pathway images (run ChatGPT-4o API 50 times).

**Supplementary Table 9**. Two-step prompt-based image analyses over 18 plant phosphorylation pathway images (run ChatGPT-5 API 10 times).

**Supplementary Table 10**. Two-step prompt-based image analyses over 18 plant phosphorylation pathway images (run Gemini API 10 times).

**Supplementary Table 11**. Impact of image complexity by the cropping test (on image 12).

**Supplementary Table 12**. Impact of randomized gene name substitution on model performance.

**Supplementary Table 13**. Prompt testing by adding a random ASCII code (run 10 times on image 6).

**Supplementary Table 14**. Evaluation using few-shot prompts (based on images 11 and 12 as references) across the remaining ten pathway images (images 1-10).
