# Supplementary material for: Multimodal knowledge expansion widget powered by plant protein phosphorylation database and ChatGPT
Source: Front Bioinform. 2025 Oct 15;5:1687687. doi: 10.3389/fbinf.2025.1687687 (PMC12568720; doi:10.3389/fbinf.2025.1687687)

Supplementary Figure 1. Illustration of the global entry prompt using the ChatGPT-4o (OpenAI) web interface. General user queries are automatically normalized into phosphorylation-specific questions through prompt engineering.

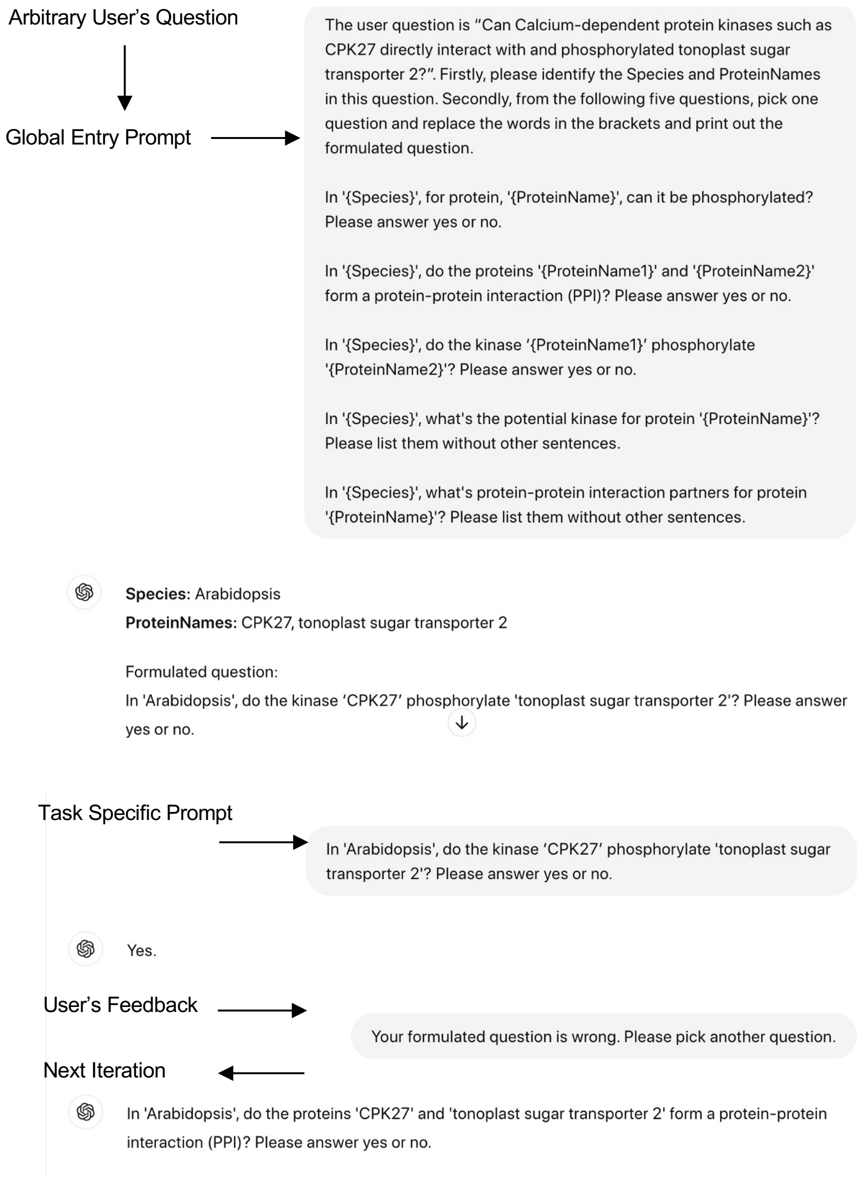

Supplementary Figure 2. Phosphorylation-specific tasks and evaluation results using P3DB datasets and ChatGPT5 API. (A) Precision scores for the “Is this protein phosphorylated?” task. (B) Precision scores for the same task in major plant species (Arabidopsis, soybean, maize, rice), using UniProt IDs and full protein names from randomized P3DB data. (C) Precision scores across kinase families for the “Does this kinase phosphorylate the substrate?” task, using KiC-assay data from Arabidopsis. (D) Precision scores for Arabidopsis protein-protein interaction (PPI) questions, evaluated using protein names.

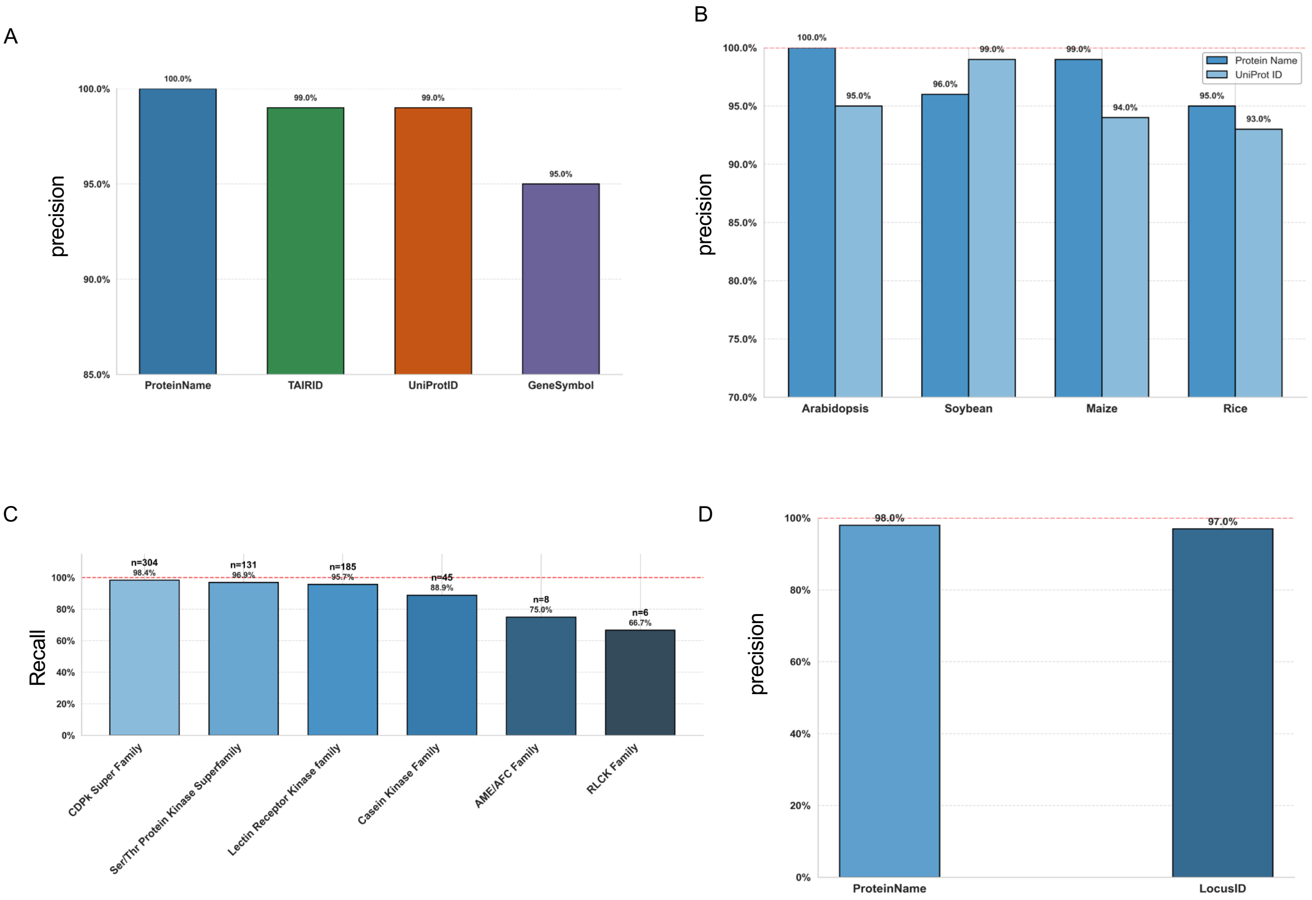

Supplementary Figure 3. Phosphorylation-specific tasks and evaluation results using P3DB datasets and Gemini API. (A) Precision scores for the “Is this protein phosphorylated?” task. (B) Precision scores for the same task in major plant species (Arabidopsis, soybean, maize, rice), using UniProt IDs and full protein names from randomized P3DB data. (C) Precision scores across kinase families for the “Does this kinase phosphorylate the substrate?” task, using KiC-assay data from Arabidopsis. (D) Precision scores for Arabidopsis protein-protein interaction (PPI) questions, evaluated using protein names.

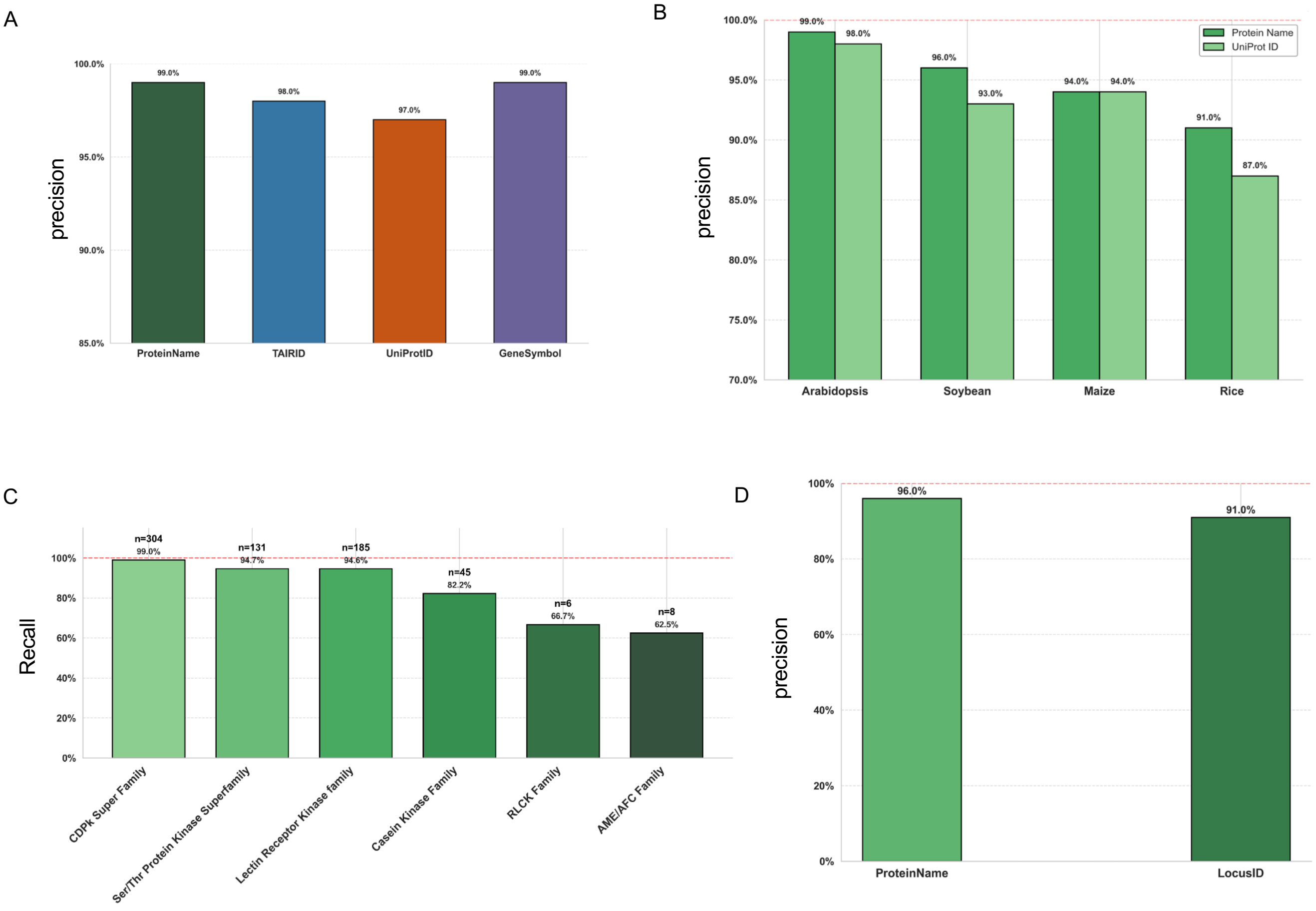

Supplementary Figure 4. ChatGPT5 image processing results for 18 pathway diagrams using two-step prompt approach. (A) Distribution of average performance scores across all images by two-step prompt method. (B) Distribution of the standard deviation of performance scores for the two-step prompt. Box plots in (C-F) showing performance distribution across 10 trials for each image. (C) PRGP (precision of regulatory gene pairs), (D) RRGP (recall of regulatory gene pairs), (E) ART (accuracy of regulatory types), and (F) APE (accuracy of phosphorylation event identification).

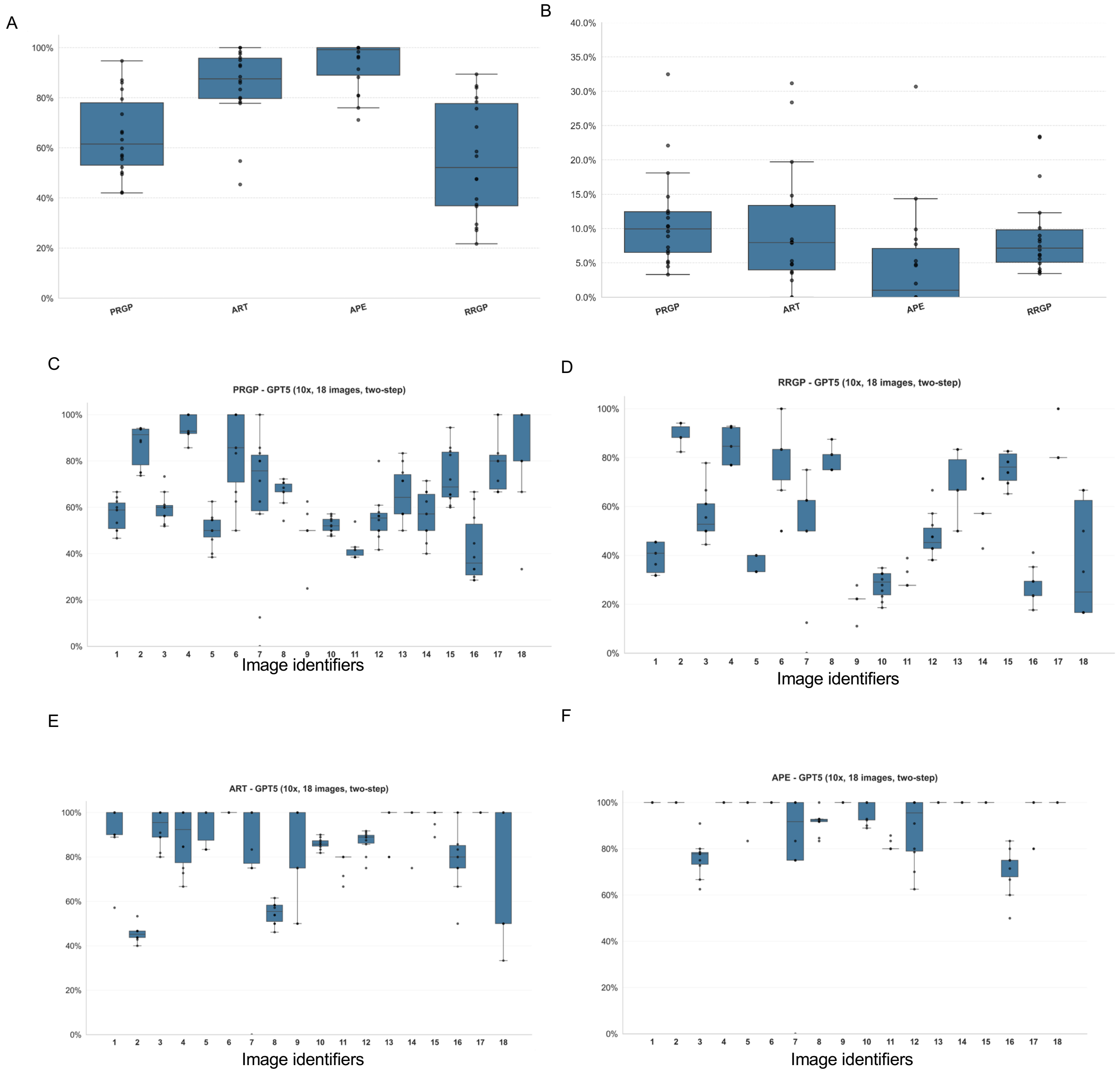

Supplementary Figure 5. Gemini image processing results for 18 pathway diagrams using two-step prompt approach. (A) Distribution of average performance scores across all images by two-step prompt method. (B) Distribution of the standard deviation of performance scores for the two-step prompt. Box plots in (C-F) showing performance distribution across 10 trials for each image. (C) PRGP (precision of regulatory gene pairs), (D) RRGP (recall of regulatory gene pairs), (E) ART (accuracy of regulatory types), and (F) APE (accuracy of phosphorylation event identification).

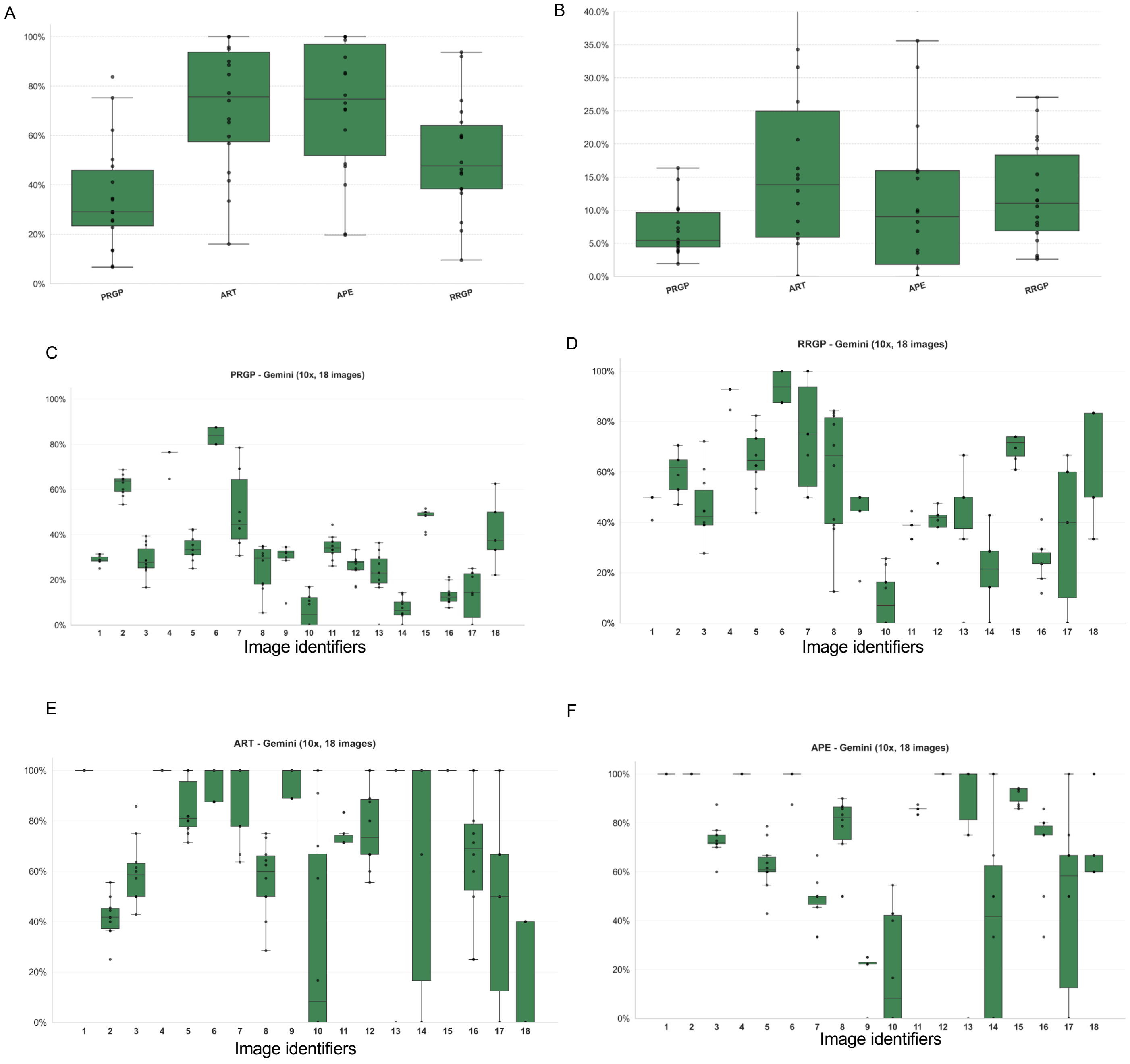

Supplement: Supplementary file 16 [file Image1.pdf]
